# Supplementary material for: A DNA G-quadruplex/i-motif hybrid
Source: Nucleic Acids Res. 2019 Nov 14;47(22):11921–30. doi: 10.1093/nar/gkz1008 (PMC7145706; doi:10.1093/nar/gkz1008)
Supplement: gkz1008_Supplemental_File [file gkz1008_supplemental_file.pdf]

Supporting Information for:

A DNA G-quadruplex/i-motif hybrid

Betty Chu, Daoning Zhang, and Paul J. Paukstelis*

University of Maryland
Department of Chemistry and Biochemistry,
Center for Biomolecular Structure and Organization
College Park, MD 20742, United States

Correspondence:

Paul J. Paukstelis
paukstel@umd.edu

Supplemental Methods

A combination of 2D-NOESY and 2D-TOCSY spectra of the 11-mer, d(CCAGGCTGCAA), was used to generate proton assignments. First, the characteristic cytosine H5/H6 NOE signals were identified in the 2D-TOCSY spectrum, allowing cytosines to be distinguished from the other three nucleotides. Next, identification of NOEs between nucleobase protons and sugar H1' and H2' protons in the 6.5-8.0 ppm region established sequential connectivity between multiple residues. We observed more signals than expected, indicating the presence of multiple conformations. In addition, overlapping resonances in this region complicated the assignment.

Nonetheless, the initial assignments were internally consistent and were verified through proton signals in the 5-6 ppm range. Finally, imino signals were assigned based on NOEs between the protons on its own ring and those from their base pairing partner. Exchangeable proton signals were confirmed from NOESY and TOCSY spectra collected on the 100% D_2O sample.

Table S1. Data Collection and Refinement Statistics.*

	**Native**	**U7-Br Derivative**	**C9-Br Derivative**
**PDB ID**	6TZQ	6TZR	6TZS
**Sequence**	d(CCAGGCTGCAA)	d(CCAGGCU^{Br}GCAA)	d(CCAGGCTGC^{Br}AA)
**Beamline**	NE-CAT 24-ID-C	NE-CAT 24-ID-C	SER-CAT BM22
**Data Collection**			
Space Group	$P3_221$	$P3_221$	$I4_122$
Cell Dimensions			
a, b, c (Å)	37.38, 37.38, 98.65	37.10, 37.10, 98.31	51.52, 51.52, 112.95
α, β, γ (°)	90, 90, 120	90, 90, 120	90, 90, 120
Resolution (Å)	49.33 – 2.29 (2.37 – 2.29)	32.77 – 2.40 (2.49 – 2.40)	55.80 – 2.60 (2.72 – 2.60)
R_{meas} (within I+/I-)	0.142 (1.708)	0.083 (0.917)	0.177 (1.889)
R_{meas} (all I+ and I-)	0.142 (1.702)	0.090 (0.917)	0.182 (1.895)
R_{pim} (within I+/I-)	0.045 (0.543)	0.037 (0.399)	0.072 (0.712)
R_{pim} (all I+ and I-)	0.035 (0.400)	0.032 (0.298)	0.0058 (0.526)
No. of unique	3955 (373)	3399 (358)	2540 (294)
I / σ I	8.2 (1.4)	11.0 (1.8)	11.5 (1.2)
Completeness (%)	99.9 (100.0)	99.9 (100.0)	100.0 (100.0)
Multiplicity	17.4 (18.0)	8.9 (9.4)	12.2 (12.7)
Wavelength (Å)	0.9196	0.9196	0.9187
**Phasing**			
Atom/Sites		Br/2	Br/2
CFOM from SHELX (1)		72.7	84.4
**Refinement**			
Resolution (Å)	32.88 – 2.29 (2.35 – 2.29)	32.15 – 2.40 (2.46 – 2.40)	46.78 – 2.60 (2.67 – 2.60)
No. reflections	3560	3040	2272
No. reflections used in R_{free} Test Set	373	338	254
R_{work}**	0.2223	0.2121	0.2665
R_{free}**	0.2551	0.2538	0.3165
$R_{complete}$**	0.2551	0.2516	0.3156
Total No. of atoms	446	445	453
Average B-factors (Å^2)	71.434	79.866	46.873
RMS deviations			
Bond lengths (Å)	0.0055	0.0062	0.0076
Bond angles (°)	1.5652	1.6400	1.6148

*Values in parentheses correspond to the high-resolution shell.
**R_{work}, R_{free}, and $R_{complete}$ values are from 10-fold cross-validation in PDB-REDO (2).

Table S2. Base pair and base pair step parameters, calculated with x3DNA-DSSR (3), for the helical core region of the d(CCAGGCTGCAA) tetramer.

Local Base Pair Parameters

	Base Pair	Shear	Stretch	Stagger	Buckle	Propeller	Opening
**1**	C1-C1	1.97	1.50	-0.04	-2.24	-0.02	177.33
**2**	C9-C9	2.09	1.33	-0.01	-2.36	6.52	177.39
**3**	C2-C2	2.04	1.44	0.16	-0.24	-3.42	179.23
**4**	A3-A3	-4.16	1.39	0.69	8.05	10.39	-112.03
**5**	G4-G5	-1.45	-3.52	0.55	-16.11	10.74	87.68
**6**	G5-G4	1.65	3.38	-0.05	8.71	0.68	-89.11
**7**	A3-A3	-4.16	1.39	0.69	8.05	10.40	-112.04
**8**	C2-C2	2.04	1.44	0.16	-0.24	-3.42	179.23
**9**	C9-C9	2.09	1.33	-0.01	-2.36	6.51	177.40
**10**	C1-C1	1.97	1.50	-0.04	-2.25	-0.02	177.32

Local Base Pair Step Parameters

	Step	Shift	Slide	Rise	Tilt	Roll	Twist
**1**	CC/CC	-2.48	2.23	0.09	126.28	126.29	-136.14
**2**	CC/CC	1.70	-2.59	0.02	148.83	95.33	-82.42
**3**	CA/AC	-3.99	-5.66	1.62	-169.56	54.42	21.15
**4**	AG/GA	1.98	3.58	-3.52	-0.89	-2.11	-95.65
**5**	GG/GG	-2.39	-2.26	-1.95	86.27	-155.46	39.34
**6**	GA/AG	-0.05	3.29	3.15	-1.71	-0.38	-85.84
**7**	AC/CA	3.99	5.66	-1.62	169.56	-54.41	-21.14
**8**	CC/CC	-1.70	2.59	-0.02	-148.82	-95.33	82.37
**9**	CC/CC	2.48	-2.23	-0.08	-126.28	-126.28	136.23

Table S3. Chemical shift values (in ppm) of ^{1}H assignments obtained from 2D-NOESY/TOCSY NMR spectra of the 11-mer, d(CCAGGCTGCAA).

	Imino	Amino	H8/H6	H5/Methyl	H1'	*H2', H2''	H3'	H4'	*H5', H5''
**C1**	-		7.39	5.53	5.75	1.73, 1.66	4.34	3.89	3.48, 3.43
**C2**	12.66	8.23, 6.85	7.40	5.65		2.05, 1.90	4.69		
**A3a**	-	7.56, 7.49							
**A3b**	-	10.21, -	8.04	-	5.63	2.54, 2.62	4.79		
**G4**	12.89	7.45, 7.39	7.61	-	5.74	2.29, 2.62	4.79		
**G5**	12.72	7.62, 7.48	7.62	-	5.58	2.28, 2.61	4.66		
**C6**	-	6.54, 6.25	7.22	4.97	5.87	1.80, 2.22	4.54	4.08	
**T7**	13.83	-	7.03	1.41	5.52	1.65, 2.00	4.61	3.86	
**G8**	-		7.63	-	5.71	2.26, 2.34	4.66		
**C9**	-		7.19	5.58	5.66	1.49, 1.95	4.44	3.78	
**A10**	-		7.86	-	5.73	2.21, 2.35	4.63	3.99	3.74, 3.66
**A11**	-		7.98	-	5.96	2.20, 2.40	4.47	3.93	

*H2'/H2'' and H5'/H5'' protons were not stereospecifically assigned.

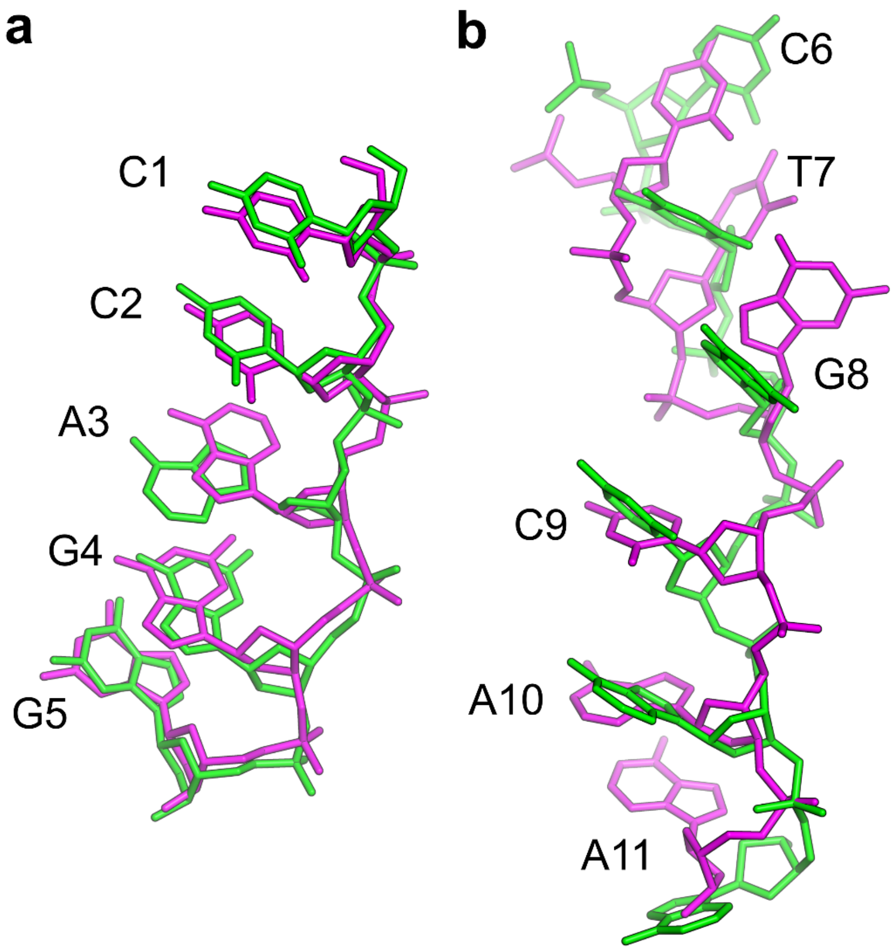

Figure S1. Structural Comparison of d(CCAGGCTGCAA) Monomers. Stick representation of the alignment between Chains A (green) and B (magenta) reveal structural similarity in residues 1-5 (a) and significant differences in residues 6-11 (b).

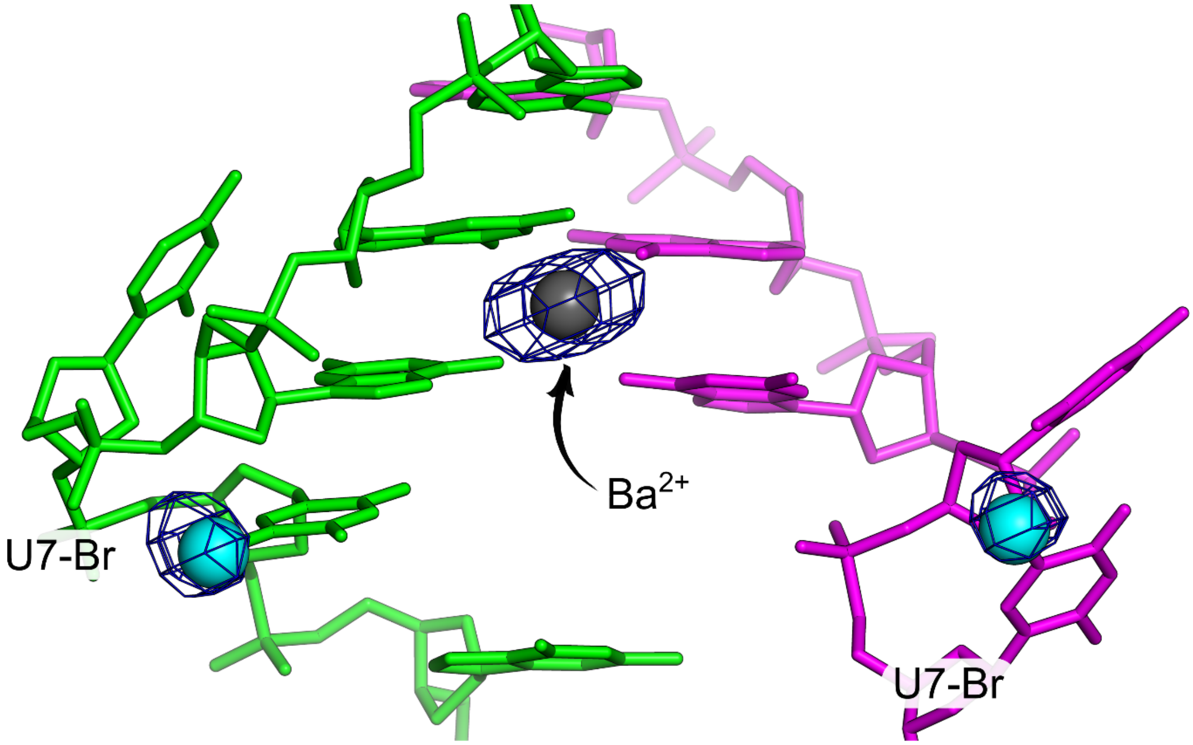

Figure S2. Anomalous Differences of the U7-Br Derivative. Anomalous difference electron density (dark blue) contoured at 6.2 σ corresponds to the bromine atoms (cyan spheres) used for phasing. A Ba^{2+} ion is shown as a gray sphere in anomalous electron density.

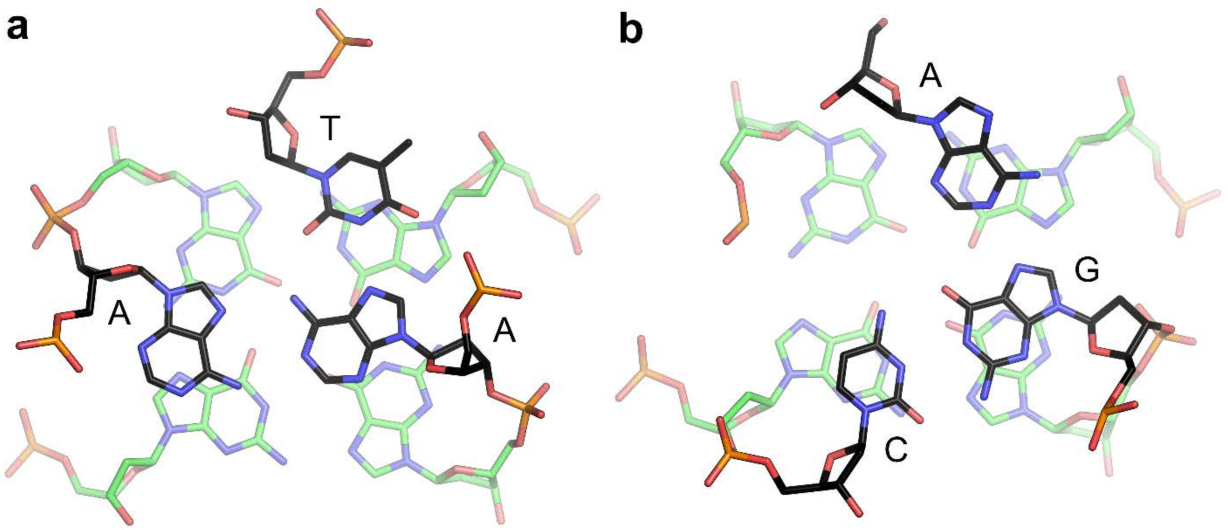

Figure S3. Structural Comparison of the Base triple/G-tetrad Stacking between the 11-mer and 2KM3. A base triple (black) stacks above a G-tetrad (green) with the *abab* topology of guanosine residues in (a) the 11-mer and (b) PDB 2KM3 (4).

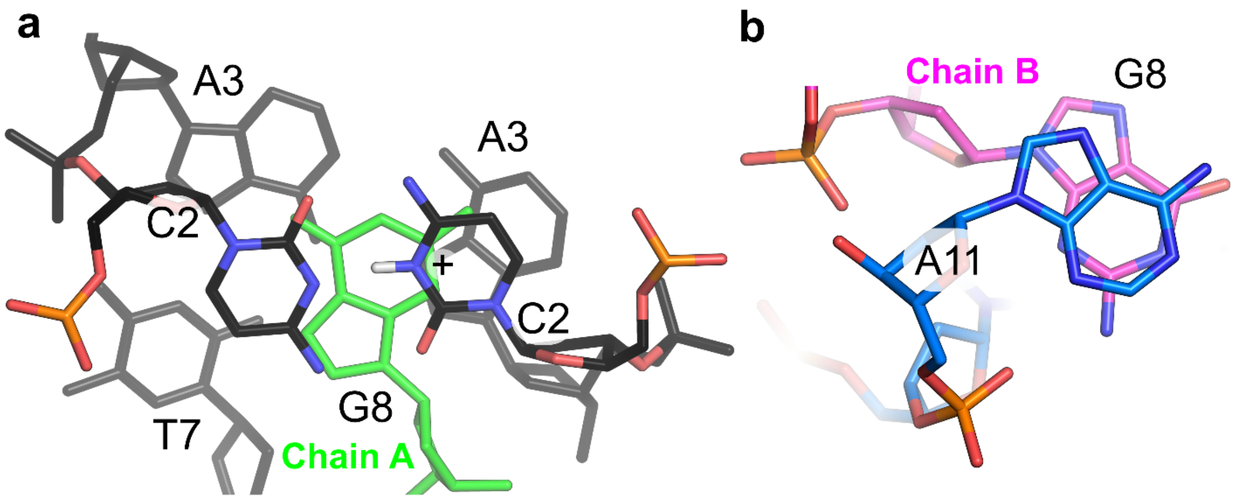

Figure S4. G8 Interactions. (a) The unpaired G8 from Chain A (green) stacks between the C2-C2$^+$ base pair and the A-A-T base triple. (b) The bulged G8 from Chain B (magenta) stacks with A11 from a symmetry-related molecule (blue).

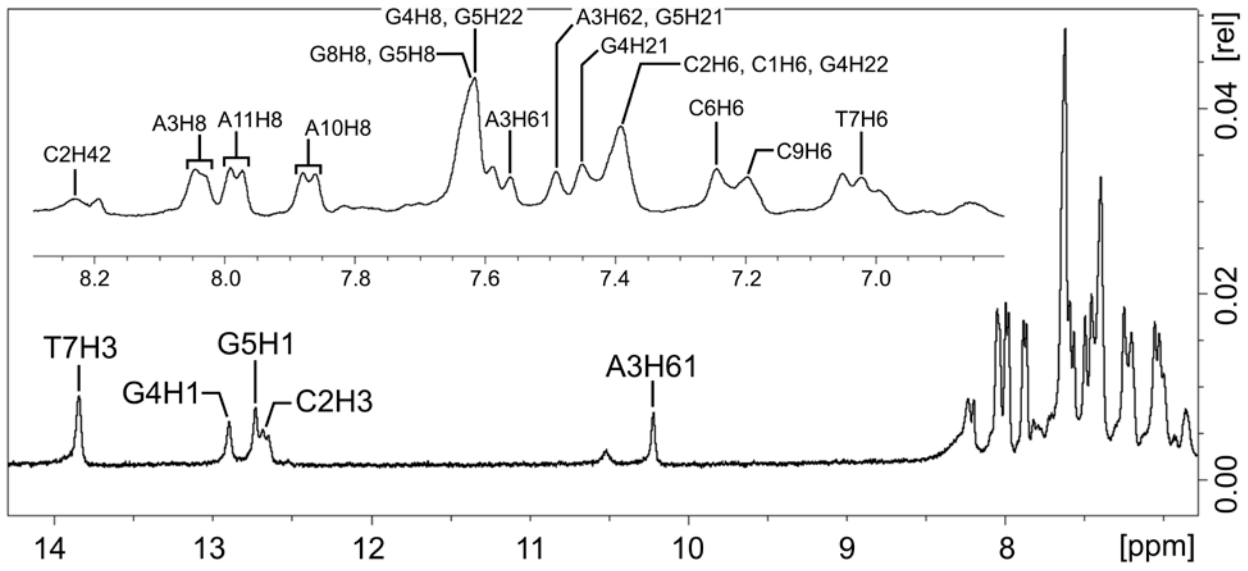

Figure S5. 1D ^{1}H NMR spectrum of the 11-mer. The assigned peaks in the imino and nucleobase (inset) regions are labeled. The C2H3 and the H8 protons of the adenosine residues show up as degenerate signals. The peak at 10.5 ppm is an artifact.

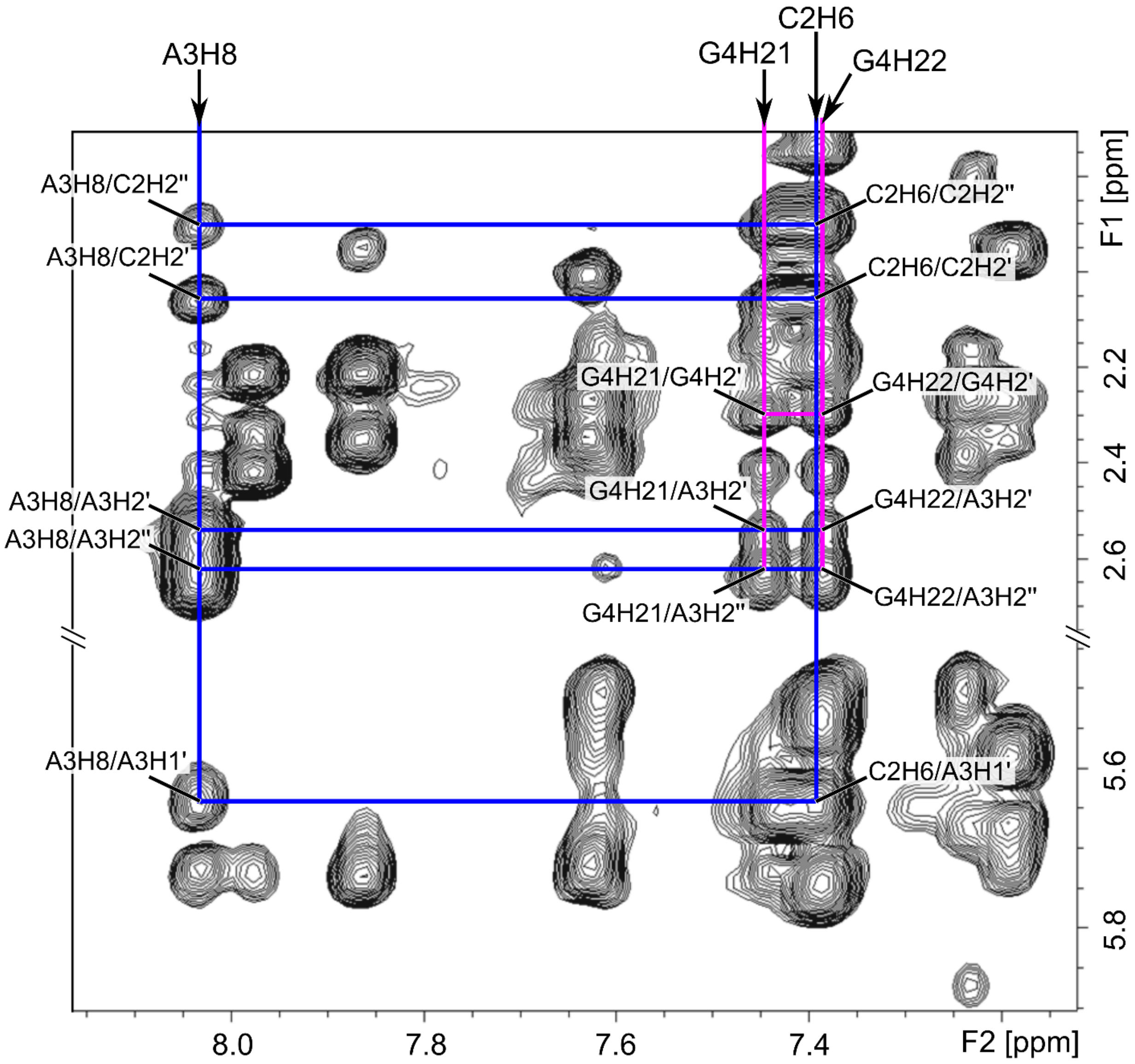

Figure S6. Regions of the 11-mer 2D-NOESY NMR spectra showing connectivity from C2 to G4. The C2H6/A3H1' and A3H8/A3H1' cross-peaks demonstrate the proximity between C2 and A3, whereas the G4H21/A3H2', G4H21/A3H2'', G4H22/A3H2', and G4H22/A3H2'' cross-peaks confirm the connectivity from A3 to G4.

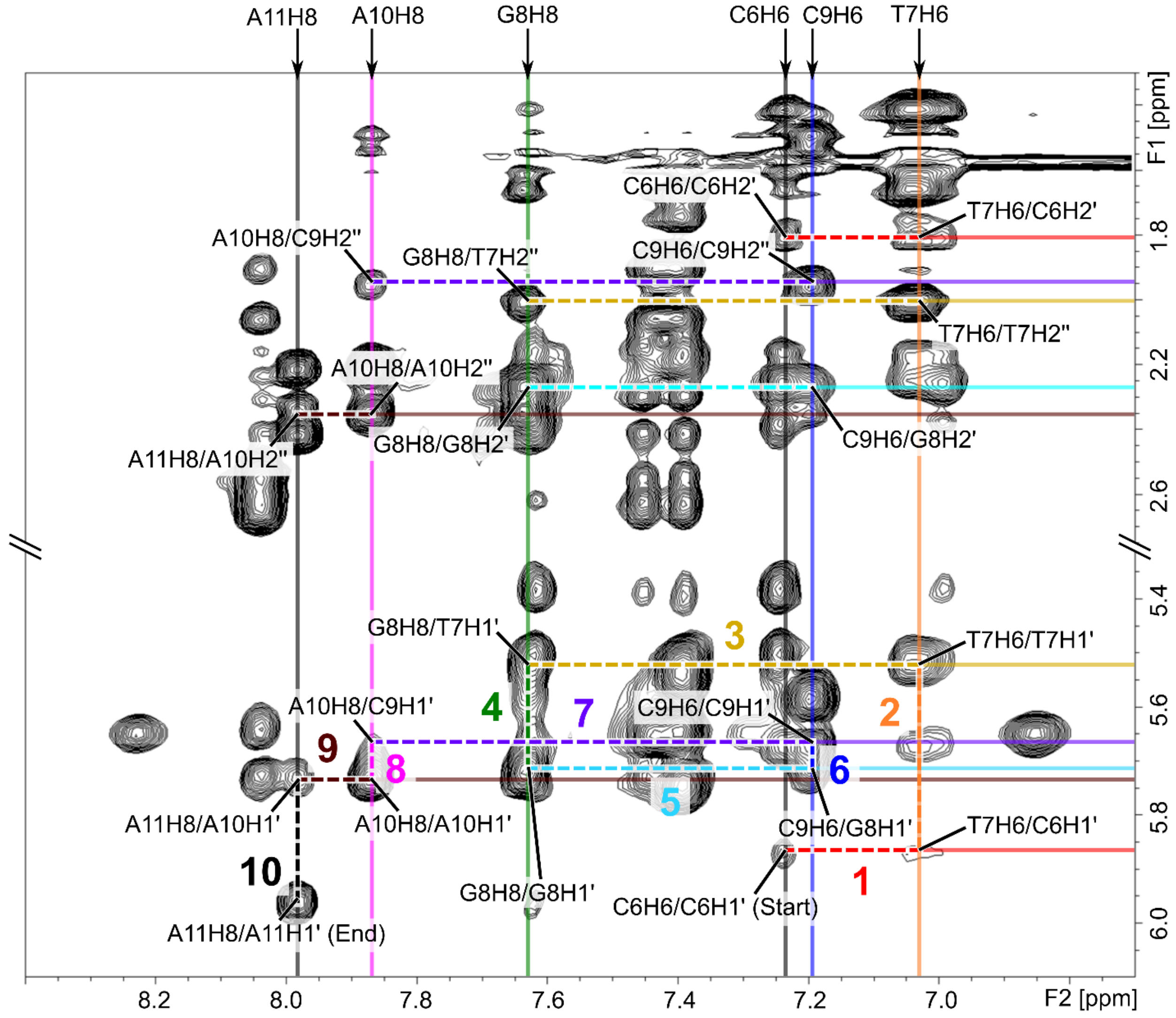

Figure S7. Sequential intra-strand connectivities for C6→T7→G8→C9→A10→A11 of the 11-mer. Cross-peaks in the non-exchangeable proton regions of the 2D-NOESY NMR spectra are labeled and indicated by intersecting lines. The sugar-to-base (H8/H6 to H1') connectivities are colored and numbered 1 – 10. NOEs to H2' or H2'' are indicated in the same color scheme and demonstrate internal consistency.

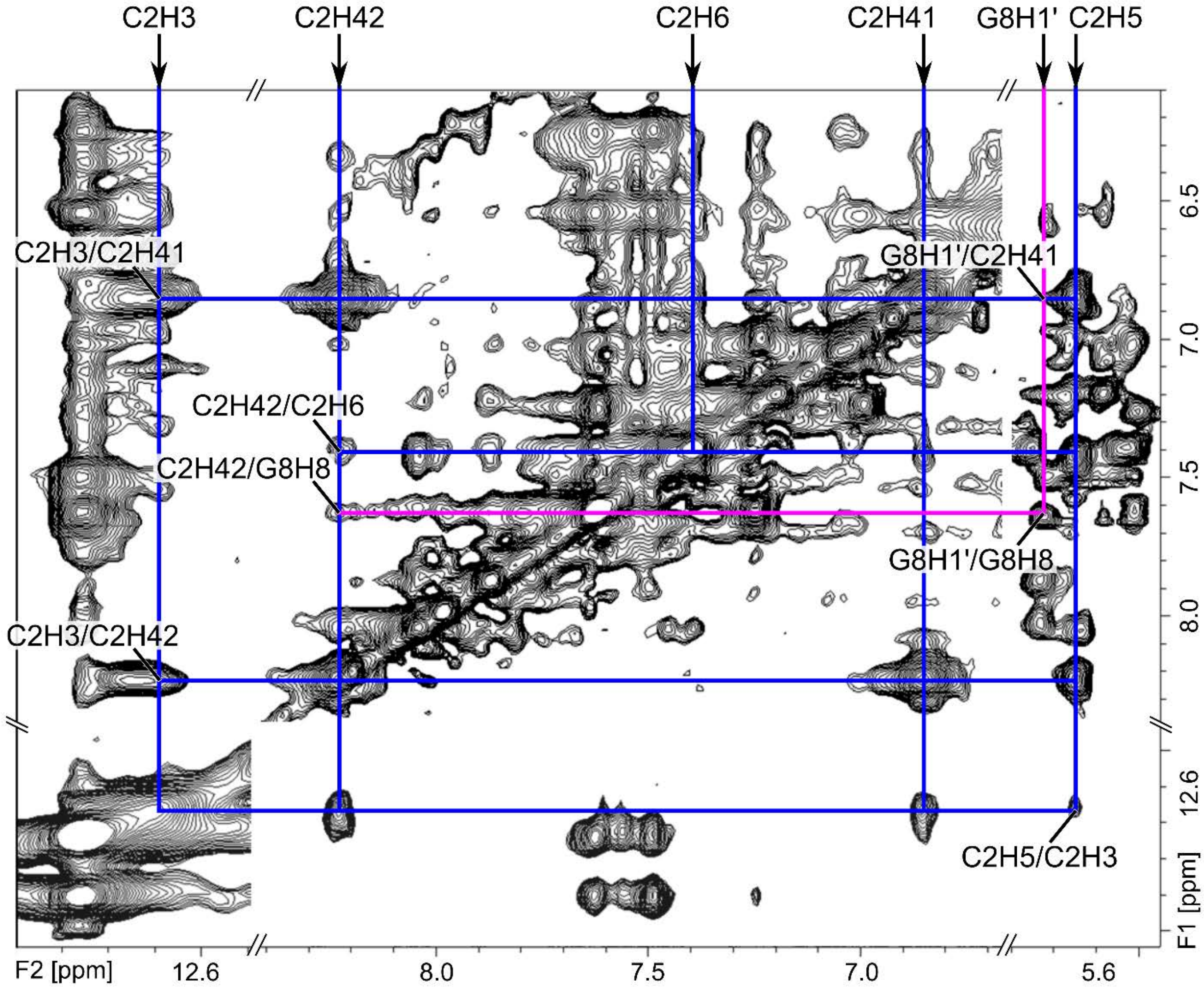

Figure S8. Regions of the 11-mer 2D-NOESY NMR spectra showing cross-peaks confirming the C2-C2^{+} base pair. The C2H3/C2H41, C2H3/C2H42, and C2H5/C2H3 cross-peaks indicate internal consistency, whereas the C2H42/G8H8 and G8H1'/G8H8 cross-peaks confirm the proximity of C2H3 and G8.

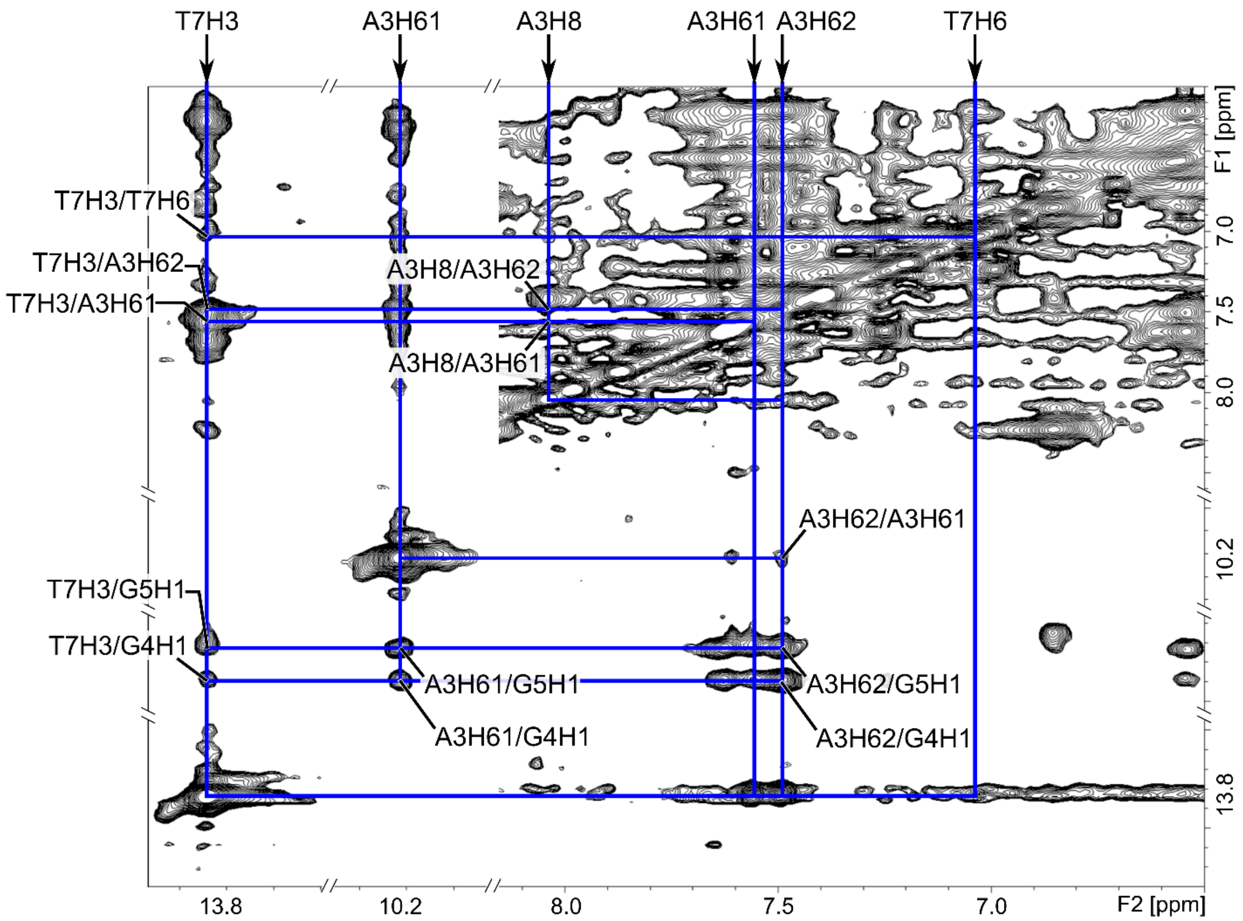

Figure S9. Regions of the 11-mer 2D-NOESY NMR spectra showing cross-peaks confirming the A-A-T base triple. The T7H3/A3H61 and T7H3/A3H62 cross-peaks confirm the T7-A3 base pair and the A3H8/A3H61, A3H8/A3H62, and A3H62/A3H61 cross-peaks from two independently assigned A3 residues provide evidence for the A3-A3 base pair. NOEs between the guanosine imino protons and the T7H3, A3H61, and A3H62 confirm the stacking between the base triple and G-tetrad.

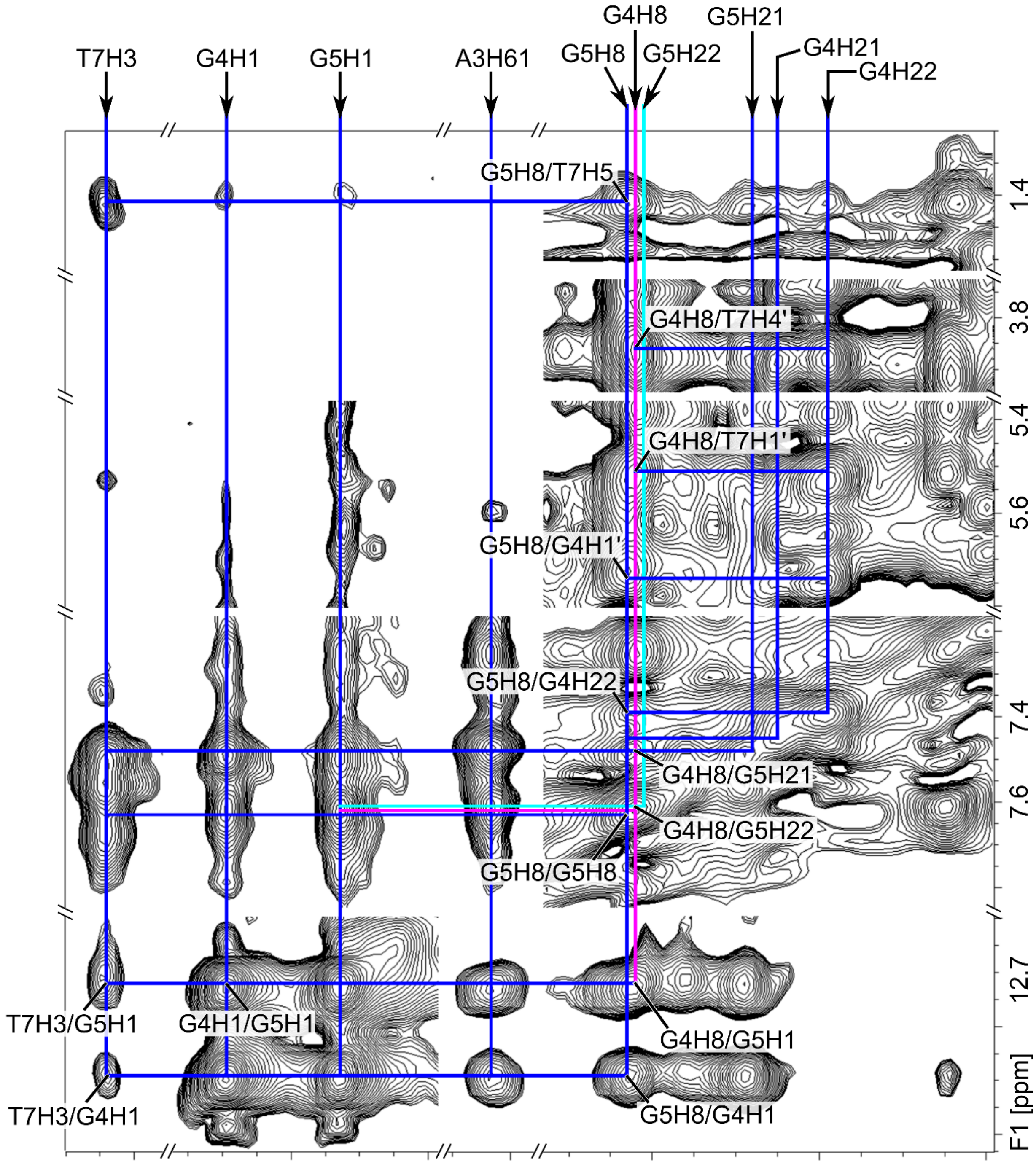

Figure S10. 2D-NOESY NMR spectra of the 11-mer showing cross-peaks confirming the G-tetrad. The G4H1/G5H1 cross-peak, in conjunction with the G4H8/G5H21, G4H8/G5H22, and G5H8/G4H22 cross-peaks, confirm hydrogen bonding between G4 and G5 through their Watson-Crick and Hoogsteen faces. The G5H8/T7H5 cross-peak, as well as resonances between G4H8 and the sugar protons of T7, confirm the arrangement of the guanosines in the G-tetrad in relation to the A-A-T base triple.

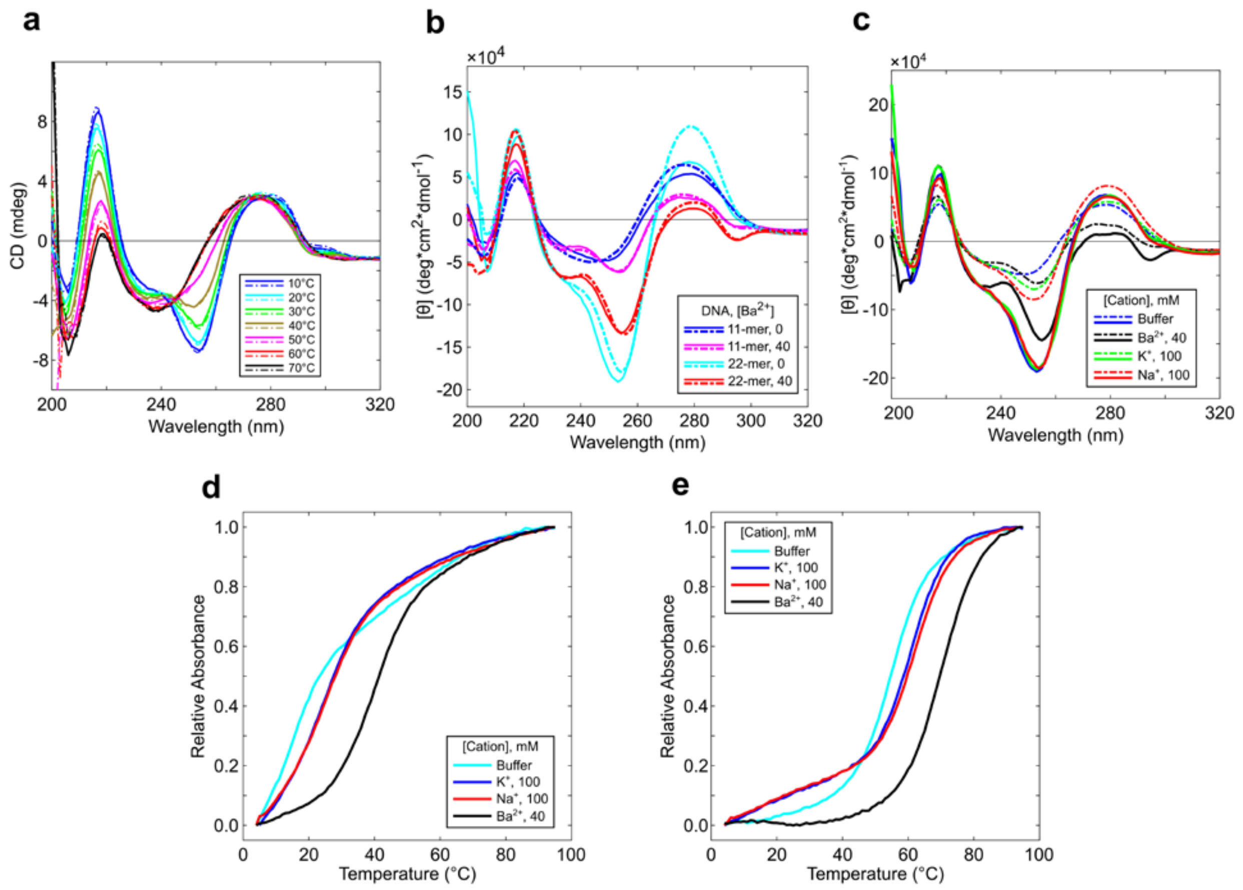

Figure S11. CD and Absorption spectra. (a) Forward (solid) and reverse (dash) CD melting curves of the 11-mer at 40 mM Ba^{2+} collected at different temperatures. (b) CD spectra of the 11-mer and 22-mer in sodium cacodylate buffer alone or supplemented with 40 mM Ba^{2+} at pH 6.0 (solid) and 7.4 (dotted). (c) CD spectra of the 11-mer (dotted) and 22-mer (solid) in sodium cacodylate buffer pH 6.0 or supplemented with 40 mM Ba^{2+}, 100 mM K^{+}, or 100 Na^{+}. Thermal denaturation curves from 4°C to 95°C of the 11-mer (d) and 22-mer (e).

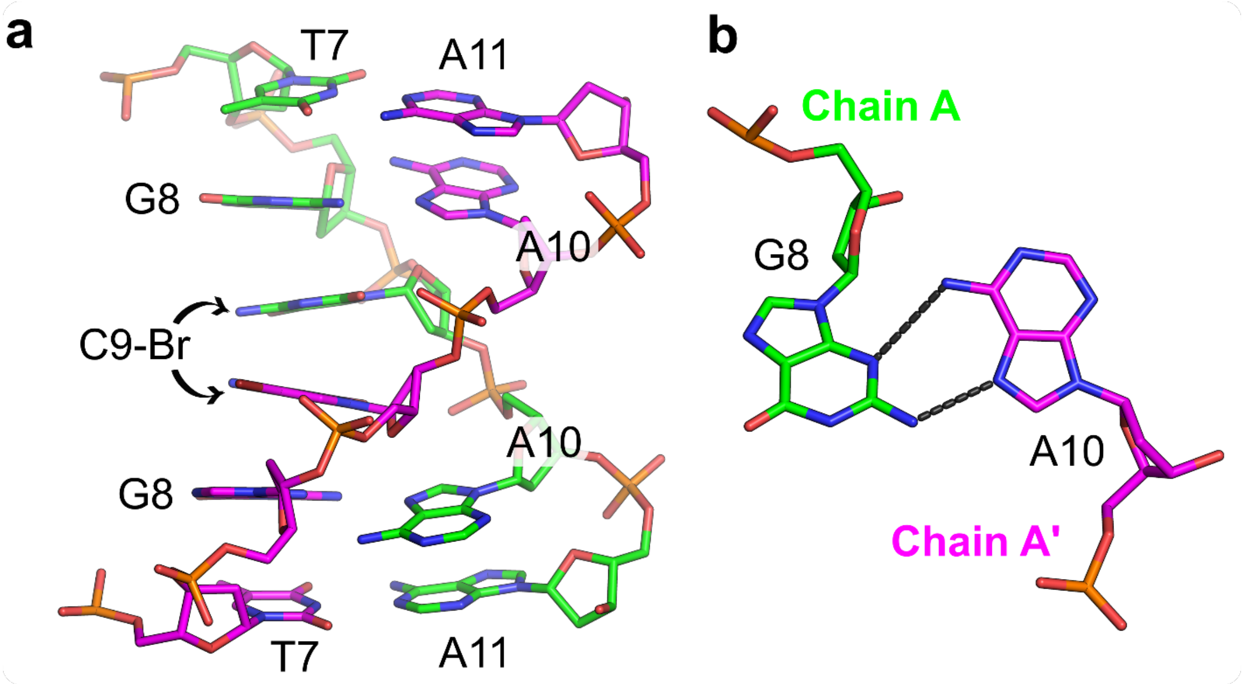

Figure S12. d(CCAGGCTGC^{Br}AA) Duplex. (a) Stick representation of the 3' antiparallel duplex formed from residues T7 through A11. (b) Residue G8 from Chain A (green) base pairs with A10 from Chain A' (magenta) through the G(N2)-A(N7) and G(N3)-A(N6) hydrogen bonds.

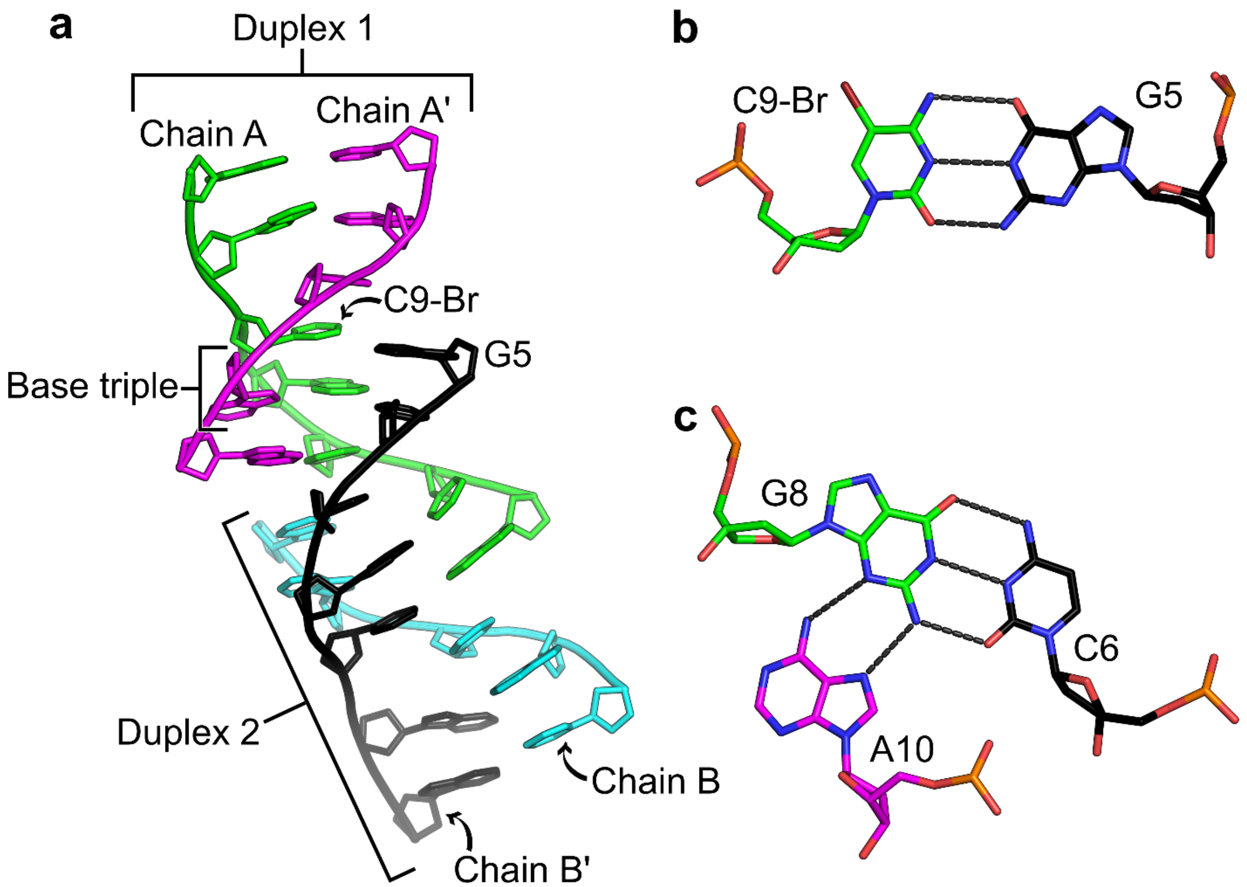

Figure S13. d(CCAGGC^{Br}TGCAA) Duplex Interactions. (a) Cartoon representation of the interactions between Duplex 1 (formed between Chains A and A') and Duplex 2 (formed between Chains B and B'). (b) Stick representation of the C9-Br-G5 base pair through standard Watson-Crick hydrogen bonding. (c) Stick representation of the C6-G8-A10 base triple. The G8-A10 base pair formed through G(N3)-A(N6) and G(N2)-A(N7) hydrogen bonding from Duplex 1 is converted into a base triple through Watson-Crick interactions with C6 from Duplex 2.

Supplementary References

1. Sheldrick, G.M. (2010) Experimental phasing with SHELXC/D/E: combining chain tracing with density modification. *Acta. Crystallogr. D. Biol. Crystallogr.*, **66**, 479-485.
2. Joosten, R.P., Long, F., Murshudov, G.N. and Perrakis, A. (2014) The PDB_REDO server for macromolecular structure model optimization. *IUCrJ.*, **1(Pt 4)**, 213-220.
3. Lu, X.J., Bussemaker, H.J. and Olson, W.K. (2015) DSSR: an integrated software tool for dissecting the spatial structure of RNA. *Nucleic Acids Res.*, **43**, e142.
4. Lim, K.W., Alberti, P., Guedin, A., Lacroix, L., Riou, J.F., Royle, N.J., Mergny, J.L. and Phan, A.T. (2009) Sequence variant (CTAGGG)n in the human telomere favors a G-quadruplex structure containing a G.C.G.C tetrad. *Nucleic Acids Res.*, **37**, 6239-6248.
